# The effectiveness of neuromuscular warm-up strategies, that require no additional equipment, for preventing lower limb injuries during sports participation: a systematic review

**DOI:** 10.1186/1741-7015-10-75

**Published:** 2012-07-19

**Authors:** Katherine Herman, Christian Barton, Peter Malliaras, Dylan Morrissey

**Affiliations:** 1 Centre for Sports and Exercise Medicine, William Harvey Research Institute, Bart's and the London School of Medicine and Dentistry, Queen Mary University of London, Mile End Hospital, Bancroft road, London, E1 4DG, UK

**Keywords:** neuromuscular training, lower limb, injuries, prevention

## Abstract

**Background:**

Lower limb injuries in sport are increasingly prevalent and responsible for large economic as well as personal burdens. In this review we seek to determine which easily implemented functional neuromuscular warm-up strategies are effective in preventing lower limb injuries during sports participation and in which sporting groups they are effective.

**Methods:**

Seven electronic databases were searched from inception to January 2012 for studies investigating neuromuscular warm-up strategies and injury prevention. The quality of each included study was evaluated using a modified version of the van Tulder scale. Data were extracted from each study and used to calculate the risk of injury following application of each evaluated strategy.

**Results:**

Nine studies were identified including six randomized controlled trials (RCT) and three controlled clinical trials (CCT). Heterogeneity in study design and warm-up strategies prevented pooling of results. Two studies investigated male and female participants, while the remaining seven investigated women only. Risk Ratio (RR) statistics indicated 'The 11+' prevention strategy significantly reduces overall (RR 0.67, confidence interval (CI) 0.54 to 0.84) and overuse (RR 0.45, CI 0.28 to 0.71) lower limb injuries as well as knee (RR 0.48, CI 0.32 to 0.72) injuries among young amateur female footballers. The 'Knee Injury Prevention Program' (KIPP) significantly reduced the risk of noncontact lower limb (RR 0.5, CI 0.33 to 0.76) and overuse (RR 0.44, CI 0.22 to 0.86) injuries in young amateur female football and basketball players. The 'Prevent Injury and Enhance Performance' (PEP) strategy reduces the incidence of anterior cruciate ligament (ACL) injuries (RR 0.18, CI 0.08 to 0.42). The 'HarmoKnee' programme reduces the risk of knee injuries (RR 0.22, CI 0.06 to 0.76) in teenage female footballers. The 'Anterior Knee Pain Prevention Training Programme' (AKP PTP) significantly reduces the incidence of anterior knee pain (RR 0.27, CI 0.14 to 0.54) in military recruits.

**Conclusions:**

Effective implementation of practical neuromuscular warm-up strategies can reduce lower extremity injury incidence in young, amateur, female athletes and male and female military recruits. This is typically a warm-up strategy that includes stretching, strengthening, balance exercises, sports-specific agility drills and landing techniques applied consistently for longer than three consecutive months. In order to optimize these strategies, the mechanisms for their effectiveness require further evaluation.

## Background

Historically, stretching as part of a warm-up strategy before exercise has been strongly advocated to prevent injury [1]. However, current evidence suggests that stretching alone may confer no injury prevention benefit [2-5]. More recently, researchers and sports medicine practitioners have developed and investigated multi-factorial neuromuscular training strategies targeting injury prevention for a variety of sports and athletic levels. The importance of musculoskeletal injury prevention is highlighted by estimates that 22 million sports injuries occur in the UK each year [6]. Furthermore, sixty to seventy percent of the population in the UK are considered to be physically inactive. Physical inactivity is currently estimated to cost the UK economy £8.3 billion per annum and is more prevalent than obesity, alcohol misuse and smoking combined [7]. It is more important than ever to encourage people to engage in some form of physical activity [8] and the recent Chief Medical Officer report 2009 described physical activity as a 'wonder drug' or 'miracle cure' with huge potential benefits [6]. However, an inevitable consequence of increasing physical activity is an increased incidence of musculoskeletal injury. To reduce the resultant personal and economic burden, there is a need for practical, time efficient and cost-effective injury prevention strategies.

Neuromuscular training programmes are hypothesized to improve joint position sense, enhance joint stability and develop protective joint reflexes, ultimately preventing lower limb injuries. Hübscher *et al. *[9] recently completed a high quality systematic review on neuromuscular training programmes for sports injury prevention. A meta-analysis indicated that multi-intervention programmes may reduce lower limb, acute knee and ankle injuries and that balance programmes may reduce ankle injuries [9]. However, the practicality of these findings for many individuals, teams and clubs may be limited due to the need for equipment purchases (for example, balance boards) and the requirement of additional training sessions to normal practice and competition. In these cases, a more practical solution would be to encompass neuromuscular training programmes which do not require additional equipment and which can be incorporated into warm-up or current routines. A number of neuromuscular warm-up strategies which fit these criteria have been proposed, evaluated and published in the literature. Just two of these programmes were included by the Hübscher *et al. *[9] systematic review. Therefore, an up-to-date systematic review of the literature related to neuromuscular warm-up strategies which can be easily incorporated into warm-up or current routines and do not require the acquisition of additional equipment is needed to further guide recommendations for effective lower limb injury prevention.

The aims of this systematic review were: (1) to evaluate the efficacy of functional neuromuscular warm-up strategies which do not require additional equipment in preventing lower limb injury in order to guide clinical and sporting practice; and (2) to identify the common elements of successful strategies in order to guide future research.

## Methods

### Search and evaluation strategy

Embase, SPORTDiscus, Google Scholar, PubMed, ISI Web of Knowledge, Scirus and PEDro were searched for articles from inception to June 2011 and updated in January 2012. Search terms included (movement training OR neuromuscular OR proprioceptive OR proprioception OR plyometric) AND (training OR program OR programme) AND prevent* AND (injury OR injuries). Limits included English language (due to the cost of translation) and human studies. The reference list of retrieved articles was manually checked for potentially relevant studies.

### Inclusion and exclusion criteria

Inclusion/exclusion criteria are shown in Table 1.

**Table 1:** Study selection criteria.

Inclusion Criteria
Those studies:
• Which investigated neuromuscular warm-up strategies without the need for additional equipment other than that readily available at training or competition venues
• for the prevention of any lower limb injury (hip, thigh, knee, ankle, leg)
• using functional training
• could be performed anywhere (for example, on-pitch)
• without the use of specialist apparatus
• easily incorporated into regular activity
• Which are detailed enough for replication
• Where injury incidence was an outcome

**Exclusion Criteria**

Those studies:
• Where the intervention is not part of a warm-up program
• Using home-based exercises due to the poor uptake and regular commitment
• Using equipment (for example, wobble board training) due to cost and availability
• Where the intervention included training outside of sporting participation sessions
• Where participants had an ongoing injury
• Using no control or comparison group
• Which were non-peer reviewed articles
• Of single participant study design

#### Quality Assessment

A modified version of the nine item van Tulder *et al. *[10] was used to assess the methodological quality of each study. The van Tulder *et al. *[10] criteria focus on the internal validity of clinical trials and recent evidence suggests it is reliable and has good face and content validity [11]. Two independent reviewers (KH and CB) scored each criterion. Any disagreement with scoring the methodological criteria was solved by consensus and a third reviewer (DM) was available if necessary, but was not needed.

### Data extraction and analysis

Details of study design, participant characteristics, interventions, statistical analysis, results and study limitations were extracted and tabulated from each included study by one reviewer (KH). Additionally, two reviewers (KH and CB) extracted data related to participant numbers and injury incidence for the various types of lower limb injuries reported. Review Manager version 5.0 was used to calculate risk ratios (RR) and their 95% Confidence Intervals (CI) for all comparisons as well as to produce forest plots to represent this data visually. The number needed to treat (NNT) was calculated only for variables producing a statistically significant RR (that is, 95% confidence intervals did not cross 1.0). Sensitivity analysis was completed to identify if the use of equipment improved injury prevention. To complete this, the effectiveness of a selection of eight studies, five randomized controlled trials (RCTs) [12-16] and three cohort studies [17-19] which utilized equipment in their programmes and, hence, were excluded from the primary review were evaluated using the same analytical approach.

## Results

### Literature search

**Figure 1:**
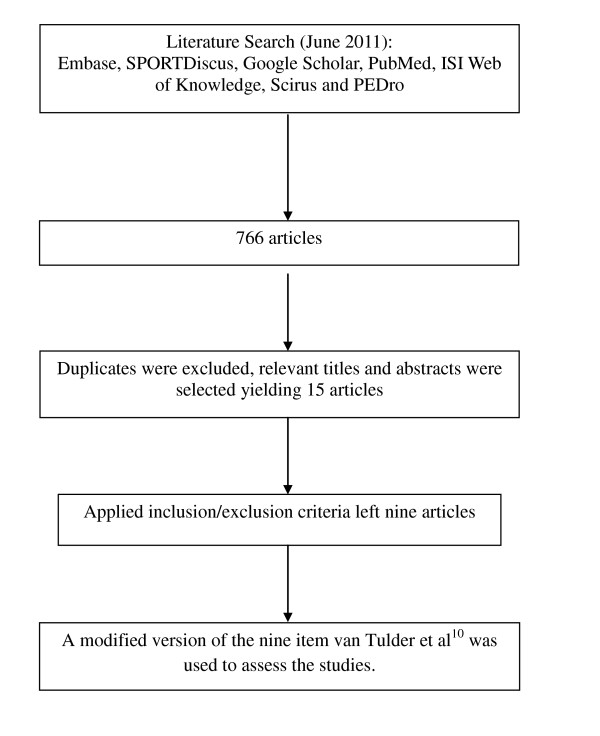
**Flowchart to demonstrate the literature search**.

The initial search identified 766 articles (Figure 1). Duplicates were excluded. Many studies were excluded because they involved use of additional equipment not readily available at training or competition venues (see Table 2). Relevant titles and abstracts were selected based on the inclusion criteria, yielding 15 articles. Application of inclusion/exclusion criteria to the full text left nine articles and excluded six articles; five studies because injury prevention was not the primary outcome [20-24] and one study because it lacked a control [25].

**Table 2:** Reasons for exclusion of studies.

Study	Reason for study exclusion
Tropp *et al. *1985	Ankle discs, orthoses used
Caraffa *et al. *1996	Balance boards used
Bahr *et al. *1997	Balance boards used
Hewett *et al. *1999	Wobble boards used
Wedderkopp *et al. *1999	Ankle discs used
Heidt *et al. *2000	Treadmill sessions implemented
Söderman *et al. *2000	Wobble boards used
Junge *et al. *2002	Not detailed enough for replication
Kaminski et al. 2003	Injury prevention not the primary outcome
Stasinopoulos *et al. *2004	Orthoses, balance boards used
Verhagen *et al. *2004	Wobble boards used
Olsen *et al. *2005	Wobble boards used
Garrick *et al. *2005	Wobble boards used
Peterson *et al. *2005	Balance boards used
Verhagen *et al. *2005	Injury prevention not the primary outcome
McKuine *et al. *2006	Wobble boards used
Mykleburst *et al. *2007	Lack of control group, mats and balance boards used
Mohammadi *et al. *2007	Orthoses, ankle weights, resistance bands, wobble boards used
McHugh *et al. *2007	Foam stability pad used
Emery *et al. *2007	Wobble boards used
Pasanen *et al. *2008	Wobble boards used
Hupperets *et al. *2008	Wobble boards used
Steffen *et al. *2008	Injury prevention not the primary outcome
Hupperets *et al. *2009	Balance boards used
Kraemer *et al. *2009	Balance boards used
Lim *et al. *2009	Injury prevention not the primary outcome
Eils *et al. *2010	Wobble boards used
Eisen *et al. *2010	Injury prevention not the primary outcome
Emery *et al. *2010	Wobble boards used
Parkkari *et al. *2011	Sticks used as part of a training approach

### Methodological quality

Table 3 shows the results of the methodological quality assessment for the nine included studies. All nine studies scored a minimum of five points on the scale indicating they were of reasonable quality [10]. The following study weaknesses were noted: failure to blind participants to the intervention [26-33], unacceptable, inadequate or absent randomization [26,27,29], failure to blind researchers to the intervention [26-28,30], no intention to treat analysis [28,30], different group values at baseline [30], high drop-out rate [32] and poor compliance [32].

**Table 3:** Assessment of methodological quality for each included study.

Methodological Quality Criteria										
**Study**	**Quality Score**	**A**	**B**	**C**	**D**	**E**	**F**	**G**	**H**	**I**

Mandelbaum *et al. *[1]	5	N	N	Y	N	Y	Y	Y	Y	N
Pfeiffer *et al. *[1]	5	N	N	Y	N	Y	Y	Y	Y	NR
Gilchrist *et al. *[1]	5	NR	N	Y	N	Y	Y	Y	Y	N
Kiani *et al. *[1]	6	N	N	Y	Y	Y	Y	Y	Y	NR
LaBella *et al. *[1]	6	Y	N	N	N	Y	Y	Y	Y	Y
Soligard *et al. *[1]	7	NR	N	Y	Y	Y	Y	Y	Y	Y
Steffen *et al. *[1]	7	Y	N	Y	Y	Y	N	Y	Y	Y
Coppack *et al. *[1]	8	Y	N	Y	Y	Y	Y	Y	Y	Y
Brushøj *et al. *[1]	9	Y	Y	Y	Y	Y	Y	Y	Y	Y

A = acceptable method of randomization, B = concealed treatment allocation, C = similar group values at baseline, D = blinded assessor, E = no or similar co-interventions, F = acceptable compliance (≥75%), G = acceptable dropout rate (≤30%), H = similar timing of the outcome assessment in all groups, I = intention to treat analysis. Y, yes; N, no; NR, not reported.

### Description of Studies

Details of each study are summarized in Table 4 including study design, participants, neuromuscular warm-up strategy evaluated, control intervention, and outcomes evaluated. Studies included an average of 1,500 participants (range 1,020 to 2,020). Two studies [33,34] investigated male and female participants, while the remaining seven investigated females only [26-32]. The age range of participants was 13 to 26 years. Five studies evaluated amateur football players [26,28,29,31,32], two studies evaluated army recruits [33,34], one study evaluated amateur football and basketball players [30] and one study evaluated amateur football, basketball and volleyball players [27]. Three studies evaluated primarily ACL injury [26-28], two studies assessed all lower extremity injury risk which included the foot, ankle, leg, knee, thigh, groin and hip [31,32], one study assessed lower extremity injuries which included knee and ankle [30], one study evaluated injuries to the knee including collateral ligament, ACL, meniscal and patella injuries [29], one study measured general overuse injuries [34] and one specifically anterior knee pain (AKP)[33]. Studies quantified injury incidence per 1,000 player hours [29,31,32], per 1,000 athlete exposures [26-28,30] and by cumulative incidence [33,34].

**Table 4:** Summary of details regarding each included study.

Study	Design	Participants	Neuromuscular warm-up program	Control Group	Outcome
Mandelbaum *et al. *[26]	CCT	1,041 female soccer players, aged 14 to 18 years	Prevent Injury and Enhance Performance Programme: three basic warm-up exercises, five stretching exercises for the trunk and lower extremities, three strengthening exercises, five plyometric exercises and three soccer-specific agility drills. Performed before matches and training, 20 minutes, for two years	Normal warm-up strategy	ACL injuries
Pfeiffer *et al. *[27]	CCT	1,439 female soccer, basketball and volleyball players, aged 14 to 18 years	Knee Ligament Injury Prevention Programme: four progressive phases of jumping and landing forwards and backwards, two- and one-footed drills, plyometric and agility training. Performed either before or after training sessions twice a week, 20 minutes, for two consecutive seasons	Normal warm-up strategy	ACL injuries
Gilchrist *et al. *[28]	RCT	1,435 female football players, average age 19.9 years	Prevent Injury and Enhance Performance Program: Three basic warm-up exercises, five stretching exercises for the trunk and lower extremities, three strengthening exercises, five plyometric exercises and three soccer-specific agility drills. Before training, 20 minutes three times a week for 12 weeks	Normal warm-up strategy	Undefined knee and ACL injuries
Kiani *et al. *[29]	CCT	1,506 female football players, aged 13 to 19 years	The 'HarmoKnee' program: warm-up, muscle activation, balance, strength, core stability exercises. Performed twice a week preseason (three months), once a week during in-season training session (six months), total duration 20 to 25 minutes	Normal warm-up strategy	All new knee injuries
LaBella *et al. *[30]	RCT	1,558 female football and basketball players, average age 16 years	Knee Injury Prevention Program: combining progressive strengthening, plyometric, balance and agility exercises. In season for one year. Total duration 20 minutes before team practices, an abbreviated version with dynamic motion exercises only before games	Normal warm-up strategy	Gradual-onset lower extremity injuries, acute-onset non-contact lower extremity injuries, non-contact knee, ACL and ankle sprains
Soligard *et al. *[31]	RCT	1,982 female football players, aged 13 to 17 years	The '11+': 10 exercises including slow running, active stretching, controlled contact, exercises for strength, balance, jumping and soccer-specific agility drills. Before training, 20 minutes, only running exercises before match, for eight months	Normal warm-up strategy	Overall and overuse lower limb injuries, groin, posterior and anterior thigh injuries, undefined knee, MTSS and undefined ankle injuries
Steffen *et al. *[32]	RCT	2,020 female football players, aged 13 to 17 years	The '11': 10 exercises for core stability, balance, dynamic stabilization and eccentric hamstring strength. Two months preseason, six months in-season before training, 20 minutes for 15 consecutive training sessions then once a week thereafter	Normal warm-up strategy	Overall lower limb injuries, groin and thigh injuries, undefined knee and ACL injuries, and undefined ankle injuries
Coppack *et al. *[33]	RCT	1,502 male and female army recruits, aged 17 to 25 years	Anterior Knee Pain Prevention Training Programme: warm-up consisted of eight exercises closed chain strengthening exercises, 10 to 14 repetitions each; warm-down involved four stretching exercises, three repetitions. Performed at each training session (mean = seven per week), 15 minutes, for 14 weeks	Normal warm-up strategy (running, stretching, strengthening)	AKP
Brushøj *et al. *[34]	RCT	1,020 female and male army recruits aged 19 to 26 years	Prevention Training Programme: Five exercises for strengthening, balance, stretching performed in three sets of five to 25 repetitions. Before military training, 15 minutes, three times a week for 12 weeks	Strategy for the upper body	Overall and overuse lower limb injuries, AKP, patella tendinopathy, ITBFS, MTSS, ankle sprain and Achilles injuries.

ACL, anterior cruciate ligament; AKP, anterior knee pain; CCT, controlled clinical trial; ITBFS, iliotibial band friction syndrome; MTSS, medial tibial stress syndrome; RCT, randomized controlled trial.

### Undefined lower limb injuries

**Figure 2:**
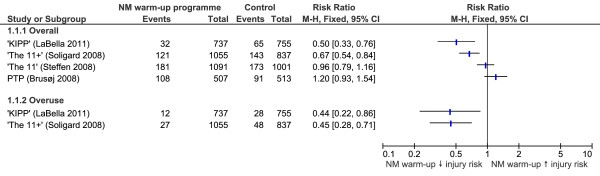
**Forest plot graph demonstrating risk ratios for the effectiveness of neuromuscular warm-up strategies in preventing undefined lower limb injuries**.

RRs for the effectiveness of neuromuscular warm-up strategies in preventing undefined lower limb injuries are shown in Figure 2. 'The 11+' [31] and 'KIPP' [30] were found to significantly reduce the risk of overall lower limb injuries (RR 0.67, CI 0.54 to 0.84, NNT 18; and RR 0.50, CI 0.33 to 0.76, NNT 24, respectively) and lower limb overuse injuries (RR 0.45, CI 0.28 to 0.71, NNT 31; and RR 0.44, CI 0.22 to 0.86, NNT 49, respectively). Similar to studies without equipment, the sensitivity analysis indicated a mixture of effective and ineffective warm-up programmes which used additional equipment.

### Hip and thigh injuries

**Figure 3:**
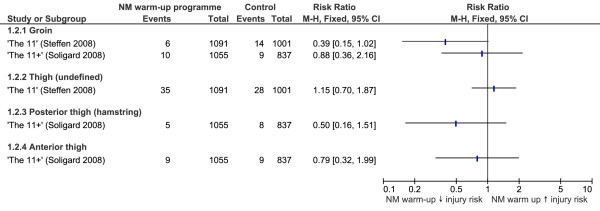
**Forest plot graph demonstrating risk ratios for the effectiveness of neuromuscular warm-up strategies in preventing hip and thigh injuries**.

None of the strategies evaluated were able to produce significant reductions in hip or thigh injuries, with calculated risk ratios shown in Figure 3. A strong trend was indicated for the 'The 11' programme [32] to reduce groin injuries (RR 0.39, CI 0.15 to 1.02, NNT 77). Similarly, the sensitivity analysis failed to identify any warm-up programmes using equipment which were able to reduce the risk of hip and thigh injuries.

### Knee injuries

**Figure 4:**
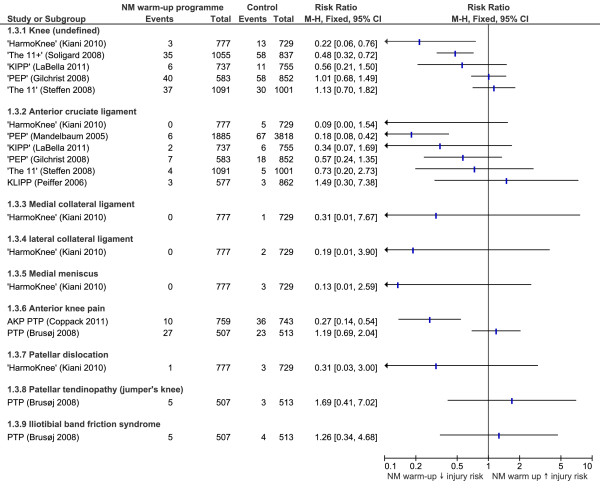
**Forest plot graph demonstrating risk ratios for the effectiveness of neuromuscular warm-up strategies in preventing knee injuries**.

RRs for the effectiveness of neuromuscular warm-up strategies in preventing knee injuries are shown in Figure 4. The 'HarmoKnee' [29] and 'The 11+' [31] programmes significantly reduced the risk of knee injuries (RR 0.22, CI 0.06 to 0.76, NNT 72; and RR 0.48, CI 0.32 to 0.72, NNT 28). Additionally, the PEP [26] strategy was the most effective at reducing ACL injuries (RR 0.18, CI 0.08 to 0.42, NNT 70). The PEP [28] also significantly reduced the risk of recurrence in those with previous non-contact ACL injuries (*P *= 0.046). The AKP PTP [33] was able to reduce the incidence of anterior knee pain (RR 0.27, CI 0.14 to 0.54, NNT 28). Similar to studies without equipment, the sensitivity analysis indicated a mixture of effective and ineffective warm-up programmes which used additional equipment to prevent knee injuries.

### Lower leg and ankle injuries

**Figure 5:**
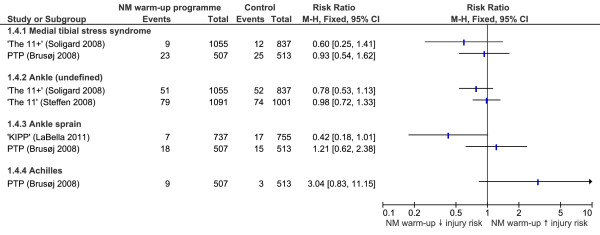
**Forest plot graph demonstrating risk ratios for the effectiveness of neuromuscular warm-up strategies in preventing lower leg and knee injuries**.

RRs for the effectiveness of neuromuscular warm-up strategies in preventing lower leg and ankle injuries are shown in Figure 5. A strong trend was indicated for the 'KIPP' [30] strategy to reduce non-contact ankle sprains (RR 0.42, CI 0.18 to 1.01, NNT 77). However, none of the neuromuscular warm-up programmes evaluated produced a significant reduction in lower leg or ankle injuries. Contrary to this, five of the eight programmes using equipment which were evaluated in the sensitivity analysis significantly reduced the risk of ankle injury. Additional equipment used in successful studies included balance boards [13-15,17], sticks [16] and medicine balls [17].

## Discussion

This systematic review investigated the effectiveness of neuromuscular warm-up strategies for injury prevention. Based on available data a number of strategies appear to be effective in preventing lower limb injuries. Specifically, 'The 11+' [31] strategy may reduce overall and overuse lower limb injuries in young amateur female footballers; the 'KIPP' [30] strategy may reduce non-contact overall and overuse lower limb injuries in young amateur female basketball and volleyball players, the PEP strategy [26,28] may reduce ACL injuries in young amateur female footballers; and the AKP PTP [33] may reduce the incidence of overuse anterior knee pain in young male and female military recruits.

### Study Analysis

The quality assessment criteria revealed that the studies had various methodological weaknesses affecting their internal validity. Firstly, sample sizes were often too low to evaluate specific injuries (for example, ankle sprains). If evaluating the effectiveness of neuromuscular warm-up programmes on more specific injuries, sample size calculations prior to commencement and recruitment of larger samples are recommended. Additionally, future studies should ensure blinding of assessors, concealment of treatment allocation, intention to treat analysis and more adequate randomization procedures to reduce the impact of issues relating to internal validity. External validity was also limited, in particular the applicability of the findings to age groups other than between 13 and 26 years.

There is also a need to determine the mechanism of effectiveness of neuromuscular warm-up strategies and determine whether injury reduction is the result of each individual component or due to a combination of exercises. No studies were identified which compared two different components or combinations of neuromuscular warm-up strategies and, in general, programmes targeted varying risk factors associated with a variety of specific injuries. Addressing this through further research will enable more emphasis on effective components of injury-specific interventions and facilitate the development of more successful neuromuscular warm-up strategies for injury prevention, specifically in reference to specific lower limb injuries.

There is limited homogeneity between the prevention strategies and the methods of recording injury incidence, making data pooling for meta-analysis inappropriate. Injury incidence was reported by a certified athletic trainer [28], a coach [26,27,30-32], an author [29,34] and participant self-reporting [33]. This may have led to a difference in the incidence of injury reporting due to the individuals' medical understanding of an injury. For example, the participants who are self-reporting may be less likely to complain of an injury perhaps due to a lack of medical insurance, compared to the author who, incidentally, is an orthopaedic consultant. The duration of the prevention strategies were 12 weeks [27,28,34], 14 weeks [33], eight months [31,32], nine months [29], and one [30] and two years [26]. Currently, it is unclear how these differences may have impacted outcomes. Further research is needed to determine the minimum participation period needed to provide protection against injury. The prevention strategies were not performed before every training session in the studies by Steffen *et al. *[32], Gilchrist *et al. *[28], LaBella *et al. *[30], Kiani *et al. *[29] and Brushøj *et al. *[34]. This potentially allowed other warm up strategies to confound any benefits of the neuromuscular training exercises and conversely for optimal effects not to be realized.

Adverse effects were only noted in four studies [26,28,31,33] and should be recorded more frequently in future studies. Those mentioned include muscle soreness at the introduction of the study [26], one minor hamstring strain [31] and one fractured tibia/fibula from falling over a ball while jumping over it [28]. It is important for studies to directly question participants about adverse effects so that safe, as well as effective, strategies are established.

### Total incidence of lower limb injuries

The effectiveness of three neuromuscular warm-up strategies in preventing the total number of lower limb injuries was evaluated in studies included in this review. Of these, only 'The 11+' [31] and KIPP [30] were found to be effective, both reducing the risk of undefined lower limb and overuse lower limb injuries. The two strategies found to be ineffective were 'The 11' [32] and the PTP [34]. In the case of the 'The 11' [32] lack of effectiveness may be explained by poor compliance. 'The 11' [32] was only used in 52% of training sessions, most likely because of a seven-week summer break during the study. The training may have had an additive effect that was lost when detraining over this period and the number of teams using the strategy after this period dropped from 60% to 44%. The authors concluded that better compliance was needed for sufficient training effects to reduce injuries.

The PTP [34] may have been ineffective at reducing lower limb injuries in military recruits due to the sudden increase in intensity of participants' training, the low load of the participants' training and the lack of supervision/training of the soldiers. Additionally, the strategy was of a short duration (12 weeks) and used less technically demanding exercises (including no warm-up, agility or plyometric exercises).

### Hip and thigh injuries

Hip and thigh injuries were recorded during the evaluation of two neuromuscular warm-up strategies, 'The 11+' [31] and 'The 11' [32]. Neither strategy significantly reduced hip or thigh injury rates, most likely because they were not powered to do so. Highlighting this, 'The 11' [32] programme indicated a strong trend toward reduced risk of groin injuries. This would likely have been a significant finding had more participants been recruited. In addition to study power, the components of the strategies may not have been adequate to reveal protection against hip and thigh injuries. A recent review of hamstring injury prevention demonstrates that isometric warm-up exercises, hamstring flexibility and concentric and eccentric strength training may be protective against hamstring injuries [35]. Additionally, core strength is a recognized factor in reducing the risk of injury. Evidence suggests that core muscle weakness may increase the risk of groin strain injuries [36]. Both 'The 11+' [31] and 'The 11' [32] incorporated Nordic hamstring curls for hamstring strength training and plank exercises for core stability. However, the number of repetitions or the frequency of these exercises may have been inadequate to reduce injury rate.

### Knee injuries

Knee injury rates were recorded in all of the nine studies, with six of these recording ACL injuries. Based on available data, four neuromuscular warm-up strategies were found to be effective in preventing knee injuries. These included individual studies showing 'The 11+' [31] and 'HarmoKnee' [29] programmes to reduce the risk of undefined knee injuries, the PEP to reduce significantly the risk of both ACL injuries [26] and their recurrence [28] and the AKP PTP [33] to reduce the risk of anterior knee pain development.

Success of the AKP PTP [33] may relate to strategy frequency as it was performed an average of seven times per week, totalling 105 minutes, a higher frequency compared to other studies. In comparison, the PTP [34] demonstrated no reductions in anterior knee pain, and this was used only three times a week totalling 45 minutes.

Despite investigating the same warm-up strategy (that is, the PEP strategy), Mandelbaum *et al. *[26] demonstrated a highly significant reduction in ACL injuries while Gilchrist *et al. *[28] showed only a trend toward risk reduction; a significant risk reduction in ACL injuries during practice, but the overall risk remaining unchanged. Reasons for this are likely to be the result of the study design and methodology. The study undertaken by Mandelbaum *et al. *[26] was a CCT with inherent methodological limitations, while Gilchrist *et al. *[28] performed a RCT providing gold standard evidence. In the study by Mandelbaum *et al. *[26] there was no blinding or randomization which introduced the potential for subject and allocation bias, respectively. Additionally, the authors in the study by Mandelbaum *et al. *[26] informed the intervention football clubs that they would be receiving a strategy to reduce injury and enhance performance. Participants and trainers were, therefore, not blinded and were likely to have been influenced by motivational bias. Of those remaining, one study informed participants of its purpose but did not disclose to which group they had been randomly allocated [27] while the others informed subjects of the purpose in a similar way to Mandelbaum *et al. *[26,28-34].

The PTP [34], and KLIPP [27] programmes did not convey any significant protection against knee injuries. As previously discussed, this may relate to lack of advice, sudden increase in training and less demanding exercises in the PTP programme [34]. Additionally, the strategy components did not include any running or agility drills, which provide a comprehensive warm-up, and have been included in successful strategies such as 'The 11+' [31]. The KLIPP [27] did incorporate such exercises but their study had inherent methodological limitations. This strategy had the lowest frequency (two times per week) and this may partly explain its lack of effectiveness. The most successful strategies ('The 11+' [31], PEP [26,28] KIPP [30] and AKP PTP [33]) were performed at every training/match session suggesting that the effectiveness of neuromuscular warm-up strategies may depend on a dose-response relationship.

The 'HarmoKnee' [29] programme significantly reduced the risk of knee injuries. However, findings did not indicate a significant reduction in the risk of specific injuries including ACL, MCL, LCL or medial meniscus injuries, despite the intervention group injury numbers being zero for each. This is due to the very low number of injuries identified in the control group, with the number being five or below for each of these specific injuries and NNTs ranging from 146 (ACL injuries) to 729 (medial meniscus injuries). Until larger studies are completed evaluating the 'HarmoKnee' [29] programme, these results must be interpreted with caution. Considering the high costs associated with surgery and rehabilitation following injuries such as an ACL tear, these reduced rates may still be clinically meaningful.

### Lower leg and ankle injuries

The effectiveness of four neuromuscular warm-up strategies which did not require additional equipment in preventing lower leg and ankle injuries were evaluated in studies from this review. Based on the results, no neuromuscular warm-up programme was able to reduce lower limb injury risk significantly. However, it should be considered that the KIPP [30] indicated a strong trend toward reduction of the incidence of ankle sprains, with an NNT of 77. Reasons for the prevention of ankle sprain injuries in the KIPP [30] strategy rather than the PTP [34] include the more comprehensive neuromuscular warm-up programme which took longer to perform and included lower repetitions of many more elements as well as dynamic exercises. Additionally, the study by Brushøj *et al. *[34] which evaluated the PTP programme had fundamental methodological flaws as mentioned earlier. No other strategies report significant reductions in lower leg or undefined ankle injuries. The '11+' [31] results showed a trend towards reduced risk of MTSS and undefined ankle injury; however, these were not convincing enough to conclude their effectiveness in injury prevention.

A previous systematic review comparing balance work (using balance boards) and neuromuscular exercises (without balance boards) revealed that ankle sprains were reduced by 36% and 50%, respectively [17]. Additionally, the sensitivity analysis completed in this review indicated that the addition of equipment, in particular balance boards, to warm-up programmes may be effective in reducing ankle injuries. This provides evidence that neuromuscular strategies can reduce ankle injuries. However, the practicality of these programmes may be questioned due to the need for acquisition of additional equipment requiring funding, maintenance and storage. Therefore, many sporting clubs and individuals, particularly in an amateur setting where most sports participation occurs, may consider that implementing such a programme is not worth the effort. In fact, this is the reason studies in our review were excluded if additional equipment were required. To impact on ankle injury prevention across all sports participation to a greater extent, design and evaluation of warm-up programmes which focus on dynamic balance and strengthening without the need for equipment such as balance boards is needed. If successful, this may provide a more practical and cost-effective alternative to using balance boards. Examples may include single leg balance exercises including throwing a ball with a partner and resisting a push from partners, hopping, and squat exercises including with heels raised and one leg squats.

### Recommendations

According to the present systematic review, several practical neuromuscular warm-up strategies which do not require additional equipment that is not readily available at the usual amateur competition or training venues are effective to varying degrees at preventing lower limb injuries. However, in some instances a large number of participants would need to undertake a strategy before one injury is prevented. This is the case with the PEP [26] strategy requiring 70 participants to prevent one injury. 'The 11+' [31], 'KIPP' [30] and 'AKP PTP' [33] appear to provide more reasonable NNT values, requiring less than 35 participants to undertake the neuromuscular warm-up strategy to prevent one injury. Of these strategies, the KIPP [30] and '11+' [31] strategies prevent the most injuries with NNTs for overall lower limb injuries being just 18 and 24, respectively.

Importantly, this systematic review highlights several areas that may account for significantly better injury prevention when incorporating neuromuscular warm-up strategies. These include: (1) incorporation of stretching, strengthening and balance exercises, sports-specific agility drills and landing techniques; (2) completing the strategy for longer than three consecutive months; and (3) completing of the strategy at all training sessions. In addition to these programme specifics, further evaluation of the '11+' [31] programme has highlighted the importance of compliance, with high compliance being linked significantly to reduced lower limb injury risk [37].

### Directions for Future Research

Further studies need to determine whether 'The 11+' [31], KIPP [30], 'HarmoKnee' [29], AKP PTP [33] and PEP [26,28] programmes are also effective in men, other age groups, and other sports as our review incorporated mainly women and involved only football, basketball, volleyball and military training. It is important to determine whether injury prevention programmes would also be effective if taught to older players who might possess more engrained poor motion patterns. In addition, healthcare professionals are encouraging middle-aged individuals to engage in sports and so research needs to include older individuals who are at a higher risk of sustaining an injury due to changed activity levels. It would also be beneficial to see if 'The 11+' [31], 'KIPP' [30], 'HarmoKnee' [29], PEP [26,28] strategy and AKP PTP [33] could be successfully combined to ultimately recommend a single injury prevention strategy. Finally, we need to know more about the mechanisms of injury prevention of neuromuscular warm-up strategies in order to optimize their effectiveness.

## Conclusions

The current systematic review identified five practical neuromuscular warm-up strategies which do not require additional equipment and which may effectively reduce the risk of lower limb injuries. Specifically 'The 11+' reduced overall and overuse lower limb injuries and knee injuries in young amateur female football players, the 'KIPP' reduced non-contact overall and overuse lower limb injuries in young amateur female football and basketball players, the 'HarmoKnee'[29] programme reduced the risk of knee injuries, the 'PEP' strategy reduced the risk of non-contact ACL injury in young amateur female football players and the 'AKPPTP' reduced the risk of anterior knee pain in male and female military recruits. Further research evaluating the effectiveness of these strategies in more varied populations, particularly men and older individuals is now needed. To provide the greatest potential for reduced lower limb injury rates, it is recommended that neuromuscular warm-up strategies incorporate stretching, strengthening and balance exercises, sports-specific agility drills and landing techniques, and are completed for a duration of longer than three consecutive months at all training sessions. Identification of which neuromuscular warm-up strategy components are most beneficial and the mechanisms behind their effectiveness is needed to further reduce lower limb injury risks.

## Competing interests

The authors declare that they have no competing interests.

## Authors' contributions

KH and CB were the primary initiators of the study while all authors made substantial contributions to data analysis and interpretation. All authors were involved in drafting and revising the manuscript and approved the penultimate version. The final version was approved and submitted by DM.

## Pre-publication history

The pre-publication history for this paper can be accessed here:

http://www.biomedcentral.com/1741-7015/10/75/prepub
